# Reply to Feng, “Recalculation of inhibitor concentration fold-changes for CMY-219: comments on a recent article about CMY-219 conferring resistance to ceftazidime-avibactam”

**DOI:** 10.1128/aac.00523-26

**Published:** 2026-06-24

**Authors:** Agnès B. Jousset, Delphine Girlich, Saoussen Oueslati, Aurélien Birer, Anne Delaval, Caroline Guyot, Ines Rezzoug, Cécile Emeraud, Thierry Naas, Rémy A. Bonnin, Laurent Dortet

**Affiliations:** 1 Université Paris-Saclay, Inserm, CEA, Immune Diseases, Microbiology and Innovative Therapies (IDMIT/UMRS1184), Fontenay-aux-Roses & Le Kremlin-Bicêtre, Le Kremlin-Bicêtre, France; 2 Department of Bacteriology-Hygiene, Bicêtre Hospital, Assistance Publique-Hôpitaux de Paris, Le Kremlin-Bicêtre, France; 3 French Associated National Reference Center for Antibiotic Resistance, Le Kremlin-Bicêtre & Clermont-Ferrand, France; 4 Service de Bactériologie, Groupe Hospitalier Intercommunal du Raincy-Montfermeil, Montfermeil, France; University of Pennsylvania Perelman School of Medicine, Philadelphia, Pennsylvania, USA

## REPLY

We thank Mr. Feng for his careful reading (1) of our work (2) and for emphasizing the importance of precise data reporting.

Regarding the IC₅₀ values, we acknowledge an imprecision in the wording in the manuscript. The sentence stating that “Avibactam IC₅₀ values were 27-fold higher for CMY-219 than for CMY-42” is not strictly accurate, as the fold-change was calculated using both CMY-2 and CMY-42 as reference enzymes (“comparators”), as was correctly indicated for cloxacillin. We appreciate the opportunity to clarify this point.

Concerning the numerical values, fold-changes were intentionally expressed as rounded orders of magnitude (e.g., 27-fold, 4,000-fold) to reflect the biological significance of the effect rather than exact ratios. This is particularly relevant given that IC₅₀ determinations were performed on crude extracts, which provide robust comparative trends (3), but do not achieve the level of precision obtained with purified enzymes.

Importantly, this clarification does not affect the interpretation of the data nor the conclusions of the study. The key finding of our work remains unchanged; for example, the CMY-219 variant results in reduced susceptibility to ceftazidime-avibactam, partly due to impaired inhibition by avibactam and cloxacillin.
